# Neural stem cell transplantation at critical period improves learning and memory through restoring synaptic impairment in Alzheimer's disease mouse model

**DOI:** 10.1038/cddis.2015.138

**Published:** 2015-06-18

**Authors:** J A Kim, S Ha, K Y Shin, S Kim, K J Lee, Y H Chong, K-A Chang, Y-H Suh

**Affiliations:** 1 Department of Pharmacology, College of Medicine, Neuroscience Research Institute, MRC, Seoul National University, Seoul, 110-799, Korea; 2 Department of Pharmacology, College of Medicine, Neuroscience Research Institute, Gachon University, Incheon, 405-760, Korea; 3 Synaptic Circuit Plasticity Laboratory, Department of Structure & Function of Neural Network, Korea Brain Research Institute, 61 Cheomdan-ro, Dong-gu, Daegu 701-300, Korea; 4 Division of Molecular Biology and Neuroscience, Department of Microbiology, School of Medicine, Ewha Medical Research Institute, Ewha Womans University, Seoul, 158-710, Korea

## Abstract

Alzheimer's disease (AD) is characterized by neuronal loss in several regions of the brain. Recent studies have suggested that stem cell transplantation could serve as a potential therapeutic strategy to halt or ameliorate the inexorable disease progression. However, the optimal stage of the disease for stem cell transplantation to have a therapeutic effect has yet to be determined. Here, we demonstrated that transplantation of neural stem cells into 12-month-old Tg2576 brains markedly improved both cognitive impairments and neuropathological features by reducing *β*-amyloid processing and upregulating clearance of *β*-amyloid, secretion of anti-inflammatory cytokines, endogenous neurogenesis, as well as synapse formation. In contrast, the stem cell transplantation did not recover cognitive dysfunction and *β*-amyloid neuropathology in Tg2576 mice aged 15 months when the memory loss is manifest. Overall, this study underscores that stem cell therapy at optimal time frame is crucial to obtain maximal therapeutic effects that can restore functional deficits or stop the progression of AD.

Alzheimer's disease (AD) is the most common neurodegenerative disorder, and is characterized by progressive cognitive dysfunction and memory loss that are caused by the death of nerve cells in several brain regions, including the cortex and hippocampus. Pathologically, senile plaques, including amyloid beta (A*β*) and carboxy-terminal fragments (CTFs) are derived via amyloid precursor protein (APP) proteolysis, and neurofibrillary tangles, including hyperphosphorylated tau, are two representative hallmarks of AD.^1, 2, 3^ Together with the accumulation of A*β*, local inflammation, altered hippocampal neurogenesis and synaptic loss have been correlated with cognitive deficits in AD patients.^4, 5^ However, no treatment has yet been developed that can cure or prevent the progression of dementia.

Accumulating evidence indicates that the transplantation of neural stem cells (NSCs) or bone marrow stem cells (BMSCs) into the hippocampus improves cognitive functions in AD animal models.^6, 7, 8, 9, 10, 11, 12^ The stem cell-induced functional recovery seems to be mediated by either neurotrophic factors and/or neuroprotective cytokines. For instance, genetically engineered stem cells that secrete nerve growth factor (NGF),^11, 12^ the co-administration of stem cells with brain-derived neurotrophic factor (BDNF) or grafting encapsulated vascular endothelial growth factor (VEGF) secreting cells substantially improved behavioral outcomes of AD animal models.^9, 13^ Thus, the functional effects of stem cell grafts involve the increase of several neurotrophic factors, such as BDNF, FGF2, insulin-like growth factor 1 (IGF1), NGF and VEGF.^10, 14, 15^ In contrast, BMSCs or adipose-derived stem cells (ASCs) transplantation induces microglial activation^16, 17, 18^ and the secretion of neuroprotective cytokines, leading to a decline of A*β* deposits and the restoration of memory deficits in AD mice.^18, 19^ However, the optimal stage of the disease for stem cell transplantation in AD models has yet to be determined.

In this study, we have investigated whether the transplantation of NSCs at two distinguished stages in the disease development could have different beneficial effects in AD model mice, Tg2576 mice.^20^ In this model, the over-production of A*β* begins at 6–7 months of age, and neuritic plaques with amyloid cores are formed from 9 to 12 months after birth followed by the onset of memory deficits at 12 months of age.^21, 22, 23^ NSCs were bilaterally transplanted into the dentate gyrus (DG) of the hippocampus and the third ventricle of 12-month-old (early stage) or 15-month-old (advanced stage) Tg2576 and age-matched wild-type (WT) mice. We determined whether the engrafted NSCs at two stages of the disease rescued cognitive deficits and the neuropathology of the mice.

## Results

### NSC transplantation at the advanced stages of the disease failed to restore behavioral deficits and pathology

**Figure 1:**
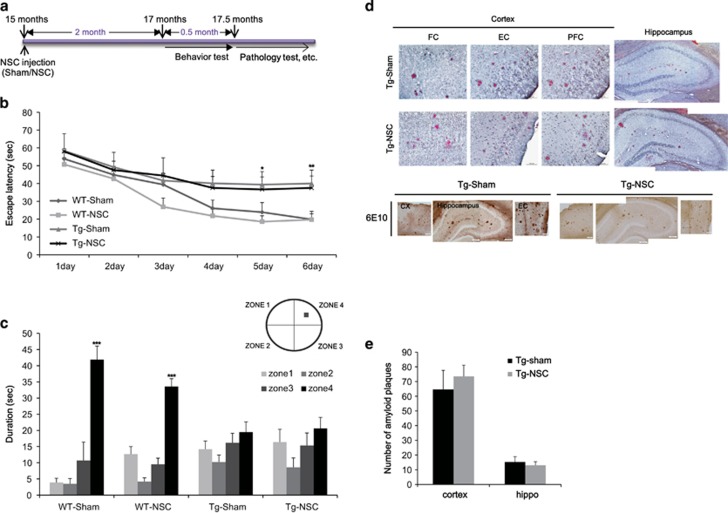
NSC transplanted at the advanced stage of AD in Tg2576 mice did not show the improvement in behavior and pathology. (**a**) We transplanted the NSC into 15-month-old Tg2576 mice, and then performed learning and memory tests 2 month after NSC treatment. After then, mice brains were isolated and the brain slices were stained with Congo red for the detection of amyloid plaques. (**b**) Mean latency in reaching the hidden platform on the spatial learning task. (**c**) The probe test represents that Tg-sham group mice cannot remember the zone 4 where the platform located during the training period. Tg-NSC group mice were not improved in the memory. (**d**) Histological analysis was carried out at the age of 17.5-month (2.5 months after the transplantation). In cortex and hippocampus, amyloid plaques were detected by immunohistochemistry using 6E10 antibody and Congo red staining. (**e**) The number of amyloid plaques was counted in brain slices containing hippocampal region of each group and calculated the average number of plaques per brain slices. There were no significant differences of amyloid plaque numbers at the cortex and hippocampal area in Tg-NSC group compared with the Tg-sham group. *P<0.05, **P<0.01, ***P<0.001 by one-way ANOVA. CX, cortex; EC, entorhinal cortex; FC, frontal cortex; PFC, piriform cortex

Initially, we transplanted NSCs into 15-month-old Tg2576 mice, and the animals were trained to locate a hidden platform in the Morris water maze at 17 months of age (Figure 1a). To examine if the spatial memory impairment in Tg2576 mice was improved by NSC transplantation, we performed water maze test. During the 6-day training period, both saline injected WT mice (WT-sham) and NSCs transplanted WT mice (WT-NSC) showed a progressive improvement of their performance to find a hidden platform (Figure 1b). The NSCs transplanted transgenic mice (Tg-NSC) group showed no significant difference in their escape latency compared with the saline injected transgenic mice (Tg-sham) group (Figure 1b). In the probe test, the WT groups spent significantly longer time in the target quadrant than the other three zones (zones 1–3) (Figure 1c). However, no significant difference between the times spent in each zone was observed in the Tg animals with or without NSC transplantation (Figure 1c). These data showed that intracerebral NSC transplantation did not improve the spatial learning impairment in 17-month-old Tg2576 mice.

To investigate whether NSC transplantation at this advanced stage affected the formation of toxic amyloid plaques, we performed immunohistochemistry with 6E10 antibody recognizing human APP, *β*-CTF and A*β* in the brains of 17.5-month-old mice. In both sham and NSC-transplanted groups of the Tg2576 mice, amyloid plaques were detected in almost all regions of the brain (Figure 1d). These data were further confirmed with an independent amyloid plaque staining, Congo red (Figure 1d). When the number of stained plaques for each group was counted and quantified, no difference was found between the sham and NSC group of Tg2576 mice in both the cortex (sham: 75±16.25; NSC: 72.33±16.25; *P*>0.05) and the hippocampus (sham: 15.66±2.90; NSC: 14±3.78; *P*>0.05; Figure 1e).

Based on these findings, we concluded that NSC transplantation in 15-month-old Tg2576 mice did not recover pathological or behavioral deficits.

### NSC transplantation at the early stages of the disease improved the learning and memory ability and reduced A*β* plaque load and tau hyperphosphorylation in Tg2576 mice

**Figure 2:**
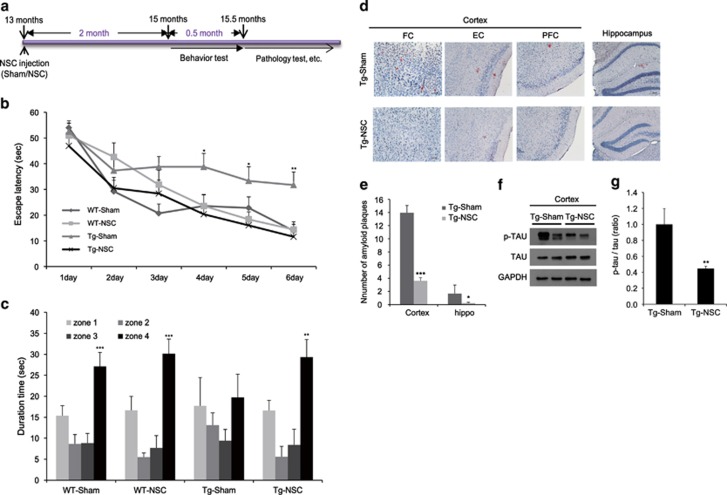
NSC transplantation at the early stage of AD attenuated the learning and memory impairment and decreased the number of amyloid plaques and tau pathology in Tg2576 mice. (**a**) We transplanted the NSC into 13-month-old Tg2576 mice, and then performed learning and memory tests 2 months after NSC treatment. After then, mice brains were isolated and the brain slices were stained with Congo red for the detection of amyloid plaques. (**b**) Training trials were conducted for 6 consecutive days. From fourth day of training trials, escape latency was significantly increased in Tg-sham group. However, the latency was decreased by Tg-NSC group compared with the Tg-sham group. (**c**) The probe test was carried out 48 h after the final training session. The times that the mice of each group stayed in zones 1–4 were compared. The time spent in the platform quadrant (zone 4) was decreased significantly in Tg-sham group. However, Tg-NSC group mice showed memory improvement compared with the Tg-sham group mice in zone 4. (**d**) Congo red staining was performed for the detection of amyloid plaques in the mouse brain. (**e**) Quantitative analysis of Congo red stained plaque number. In brains of NSC-transplanted Tg2576, A*β* deposition was significantly reduced in the cortex and hippocampus. (**f**) Phosphorylated tau and total tau were detected in cerebral cortex by western blot. (**g**) The graph represents that the amount of P-tau relative to total tau was significantly reduced in Tg-NSC group. *P<0.05, **P<0.01, ***P<0.001 by one-way ANOVA. P-TAU, phosphorylated tau

To find the optimal period for NSC transplantation, we transplanted NSCs into 13-month-old Tg2576 and age-matched WT mice, and performed the Morris water maze training at 15 months of age (Figure 2a). During the training period, the Tg-NSC group showed a significant reduction in their escape latency compared with the Tg-sham group (Figure 2b), indicating the NCS-mediated restoration in the spatial learning capability of Tg2576 mice. We found no differences between the WT-sham and WT-NSC groups (Figure 2b). In the probe test, Tg-NSC mice stayed significantly longer in zone 4 than in the other zones (zones 1–3), similar to the WT-sham or WT-NSC group (Figure 2c). In contrast, the Tg-sham mice exhibited no significant difference in the time spent in each zone (Figure 2c).

To assess their neuropathological features, we examined amyloid plaque load in the brains of 15.5-month-old WT-sham, WT-NSC, Tg-sham and Tg-NSC mice using Congo red staining. Although the Tg2576 mice showed AD-like pathologies with plaque formation in the brain, there were no plaques observed in their age-matched WT animals (Figure 2d). In the Tg-NSC group, there was a significant amyloid plaque reduction in the cortex (from 13.96±1.09 to 3.61±1.30, *P*<0.001) and the hippocampus (from 1.67±0.46 to 0.22±0.11, *P*<0.05) compared with the Tg-Sham group (Figure 2e). In the cortex, congophilic plaques were significantly decreased in the frontal, entorhinal and piriform cortex areas (frontal cortex: 73.65%, *P*<0.001; entorhinal cortex: 78.53%, *P*<0.01; piriform cortex: 65.98%, *P*<0.05 decreased separately), compared with the Tg-sham group (Supplementary Figure 2A). However, neither sham nor NSC-transplanted WT mice had congophilic plaques.

Next, we investigated the level of phosphorylated tau (p-tau), another representative pathological hallmark of AD. In the cortex of the Tg-NSC group, the p-tau levels were reduced by 55.4% (*P*<0.01), compared with the Tg-sham group (Figures 2f and g).

These data showed that the NSC transplantation at the early stages of the disease functionally recovered behavioral and pathological deficits in Tg2576 mice.

### NSC transplantation could reduce the A*β* level by altering the processing of APP and degradation of A*β*

**Figure 3:**
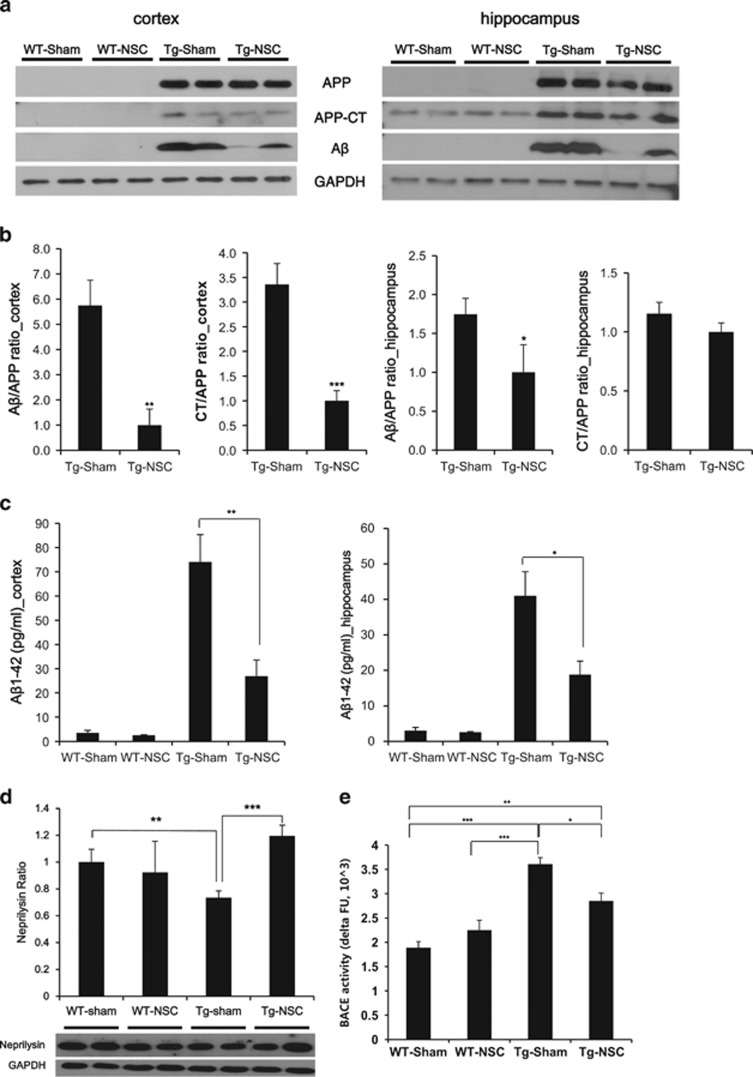
NSC transplantation influenced the processing of APP and the clearance of A*β* protein in Tg2576 mice brains. (**a**) At 2.5 months after transplantation, western blot analysis was performed with total lysates from the cortical region and hippocampal region of the brains in each group using 6E10 antibody. (**b**) A*β* and CT bands were detected and normalized by the amount of APP and GAPDH. In the Tg-sham group mice brain, a lot of A*β* and CT were produced compare with the Tg-NSC group mice. (**c**) Cortical or hippocampal A*β* level was analyzed by A*β*_1-42_ ELISA in all groups. The levels of A*β* were highly increased in the cortex and hippocampus of Tg-sham group mice compared with those of age-matched WT mice. Note that the levels of A*β* were decreased by the Tg-NSC group mice. (**d**) In cortex, the production of neprilysin was increased in 1.6-fold by NSC transplantation compared with the Tg-sham group. (**e**) Enzymatic activity of the *β*-secretase from the mice brain lysates was assessed using fluorometric reaction. *β*-Secretase activity was assessed as time passed. Twenty  minutes after adding the substrate, *β*-secretase activity showed significant difference between WT-sham, WT-NSC, Tg-sham and Tg-NSC group. In Tg-NSC group, BACE activity was decreased compared with the Tg-sham group. *P<0.05, **P<0.01, ***P<0.001 by one-way ANOVA

To identify mechanisms underlying the reduction of A*β* depositions in Tg-NSC mouse brains, the levels of A*β*, APP-CTFs and APP were quantified by western blot analysis with a 6E10 antibody. The total APP levels showed no difference between the Tg-sham and Tg-NSC groups, whereas the levels of A*β* and APP-CTFs in the cortices of the Tg-NSC mice were markedly decreased by 82.60% and 70.21%, respectively, compared with the Tg-sham animals (Figures 3a and b). A*β* levels were also reduced in the hippocampus of the Tg-NSC group (decreased by 42.79%, *P*<0.05; Figures 3a and b). Using A*β* ELISA, we measured the A*β*_1-42_ levels in all groups (Figure 3c). Consistent with the Congo red staining and the western blot results, the A*β*_1-42_ level in the Tg-NSC brain was significantly decreased by 63.62% in the cortex (from 74.04±11.26 to 26.93±6.68, *P*<0.01) and 54.21% in the hippocampus (from 40.98±6.83 to 18.76±3.85, *P*<0.05), compared with the Tg-sham group (Figure 3c). These data provide evidence that the A*β* and CTF protein levels in the brain were reduced by the NSC transplantation.

To further understand how NSC transplantation reduced A*β* levels in the Tg2576 brain, we next focused on the level of neprilysin, one of the A*β-*degrading enzymes.^24^ In Tg-NSC mice, we found a 1.6-fold increase in neprilysin compared with the Tg-sham mice (*P*<0.05, Figure 3d), indicating the enhanced degradation of A*β* by the induction of neprilysin. In addition, we evaluated the *β*- and *γ*-secretase activity involved in the APP processing. Enzymatic activity of *β*-secretase (BACE) was significantly reduced in the Tg-NSC group compared with the Tg-sham group (Figure 3e); however, *γ*-secretase activity was distinguishable but not significantly decreased (Supplementary Figure 2B).

These results suggest that intracerebrally transplanted NSCs at the early stage of AD reduce the amyloid plaque burden, as well as the levels of A*β* and CTFs by decreasing the production and increasing the clearance of A*β*.

### NSC transplantation decreased inflammatory microglial activation in the brain of Tg2576 mice

**Figure 4:**
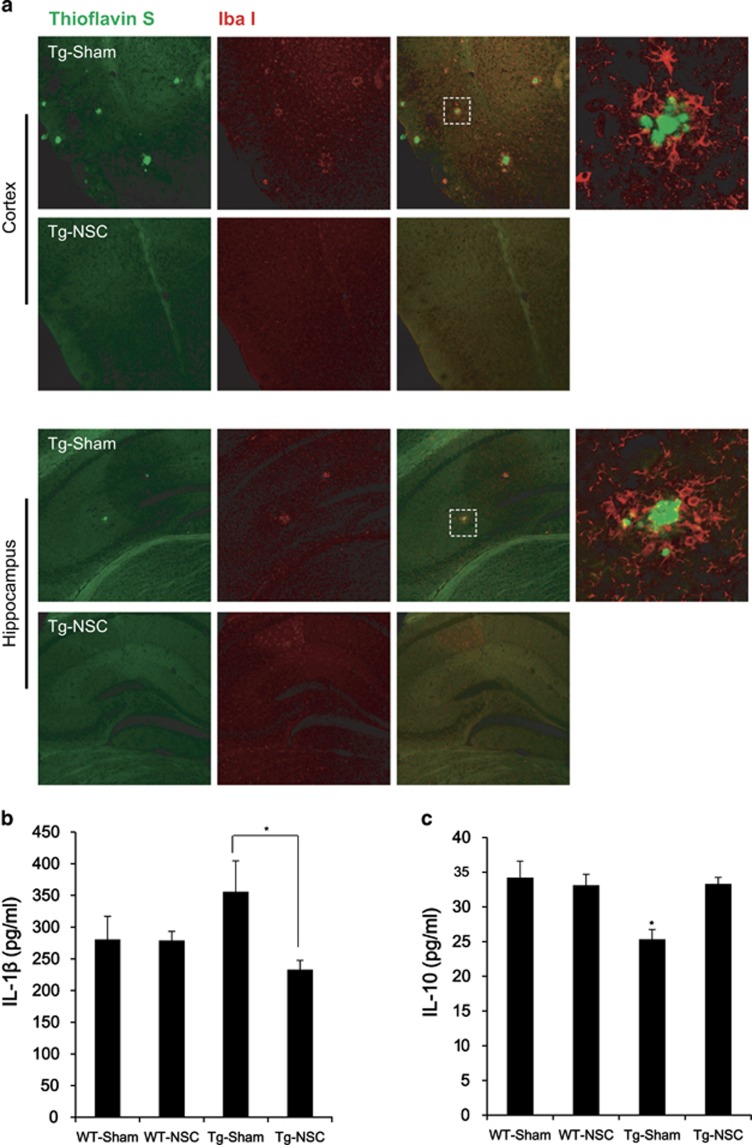
The microglial character was changed in Tg2576 mice brains. (**a**) Amyloid plaques and microglial cells in cortex and hippocampal regions were detected by the double staining of thioflavin S and IbaI antibodies. Microglial cells were gathered around the plaques. (**b** and **c**) The level of IL-1*β* or IL-10 was detected in the tissue lysates of cortical region of brain from each group by ELISA. IL-1*β*, representative inflammatory cytokine, was increased in Tg-sham group but reduced by NSC transplantation (**b**). By contrast to IL-1*β*, IL-10, representative anti-inflammatory cytokine, was increased by NSC transplantation (**c**). *P<0.05, by one-way ANOVA

Microglial activation is a hallmark of AD models and is associated with the distribution of A*β* plaques and neurofibrillary tangles, which has been correlated to degeneration, dementia progression and AD severity.^25, 26, 27^ Based on these reports, we aimed to determine whether NSC treatment altered microglial characteristics in Tg2576 mice. To examine the early stage of transplantation, mice were killed 3 weeks after the NSC transplantation. Brain sections were double-labeled using anti-Iba-1 antibody for microglia and thioflavin S staining for A*β* deposition. Confocal microscopy revealed the clustered microglial cells were in the vicinity of A*β* deposition in the brain of Tg2576 mice (Supplementary Figure 3). At 2.5 months after the NSC transplantation, inflammatory microglial activation was decreased in the brain of Tg2576 mice (Figure 4a). In the Tg-NSC mouse brain, interleukin (IL)-1*β*, a proinflammatory cytokine, was decreased (from 355.8±48.9  to 233.1±14.3 pg/ml, *P*<0.05) and IL-10, an anti-inflammatory cytokine, was increased (from 25.36±1.39  to 33.32±0.91 pg/ml, *P*<0.05) compared with the Tg-sham group (Figures 4b and c).

These data indicated that NSC transplantation may change the activated and inflammatory form of microglia to a neuroprotective form, resulting in bidirectional regulation of proinflammatory and anti-inflammatory cytokine levels.

### NSC transplantation improved endogenous neurogenesis and dendritic stability

**Figure 5:**
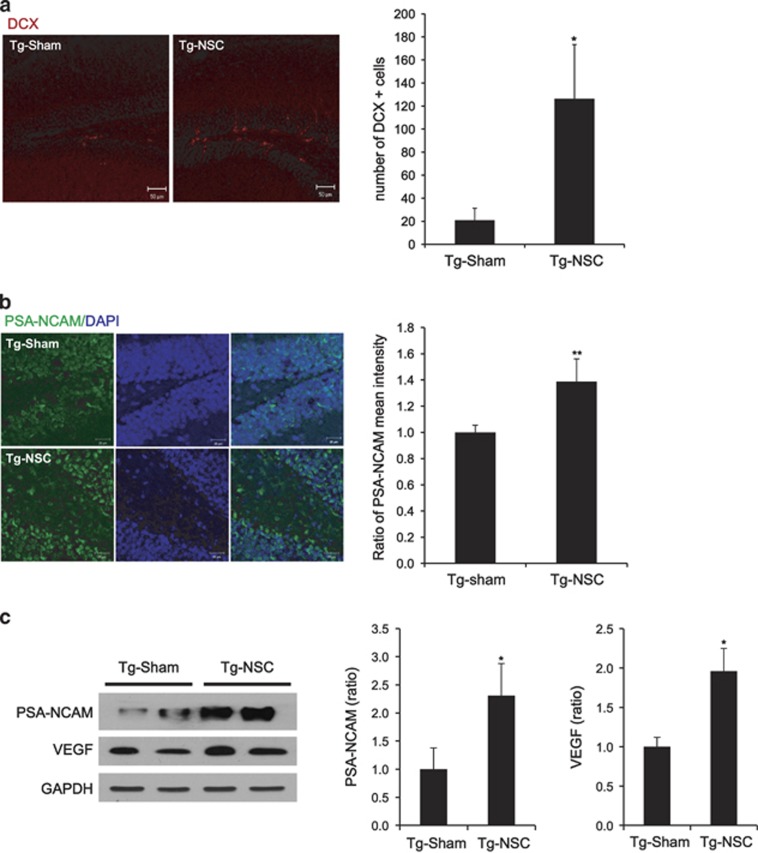
Endogenous neurogenesis was increased in the NSC-transplanted Tg2576 mice. (**a**) At 2.5 months after transplantation, endogenous neurogenesis was observed in the DG of hippocampus by immunohistochemisty using anti-DCX antibody. DCX-positive cells were calculated in mm^2^ area of DG. Graph represents that endogenous neurogenesis was enhanced by NSC transplantation. (**b**) The levels of PSA-NCAM were analyzed by immunohistochemistry. In Tg-NSC group, the levels of PSA-NCAM were increased compared with the Tg-sham group. Graph represents that PSA-NCAM was enhanced by NSC transplantation. (**c**) In Tg-NSC group, the levels of PSA-NCAM and VEGF were increased compared with the Tg-sham group. Quantitative analysis shows that NSC transplantation significantly increased the level of PSA-NCAM and VEGF expressions. *P<0.05, by one-way ANOVA

Recent reports have suggested that proinflammatory cytokines have a negative effect on neurogenesis, whereas anti-inflammatory cytokines exert the opposite effect.^28, 29^ Therefore, we reasoned that NSC transplantation would promote endogenous neurogenesis through neuroprotective factors such as anti-inflammatory cytokines. To prove this idea, we labeled the brain sections with both anti-doublecortin (DCX) and bromodeoxyuridine (BrdU) antibodies recognizing newly born neurons and counted the DCX-positive or BrdU-positive cells in the DG of the hippocampus. Remarkably, Tg-NSC mice had significantly increased number of DCX-positive neurons compared with the Tg-sham animals at 2.5 months after NSC transplantation (Tg-NSC: 126.41±46.91; Tg-sham: 20.93±10.47, *P*<0.05) (Figure 5a). We found more number of BrdU-positive neurons in DG of the NSC-treated Tg mice brains (Supplementary Figure 4). Quantitative analysis showed that the number of BrdU-positive neurons was significantly increased in the NSC-treated Tg mice (Supplementary Figure 4B).

To corroborate the effects of NSC on adult neurogenesis, we also examined the level of polysialylated-neural cell adhesion molecule (PSA-NCAM) that is highly expressed in the population of newly generated granule cell precursors, and are closely related with neurogenesis and brain plasticity.^30, 31^ PSA-NCAM was increased in the brains of the Tg-NSC group (1.4-fold increase in hippocampus; 2.3-fold increase in cortex, *P*<0.05) compared with the Tg-sham group (Figures 5b and c). Adult hippocampal neurogenesis was also induced by neurotrophic or growth factors.^32^ In accordance with the previous finding, VEGF was significantly increased by the transplantation of NSC in Tg2576 mice (2.4-fold increase, *P*<0.05; Figure 5c).

**Figure 6:**
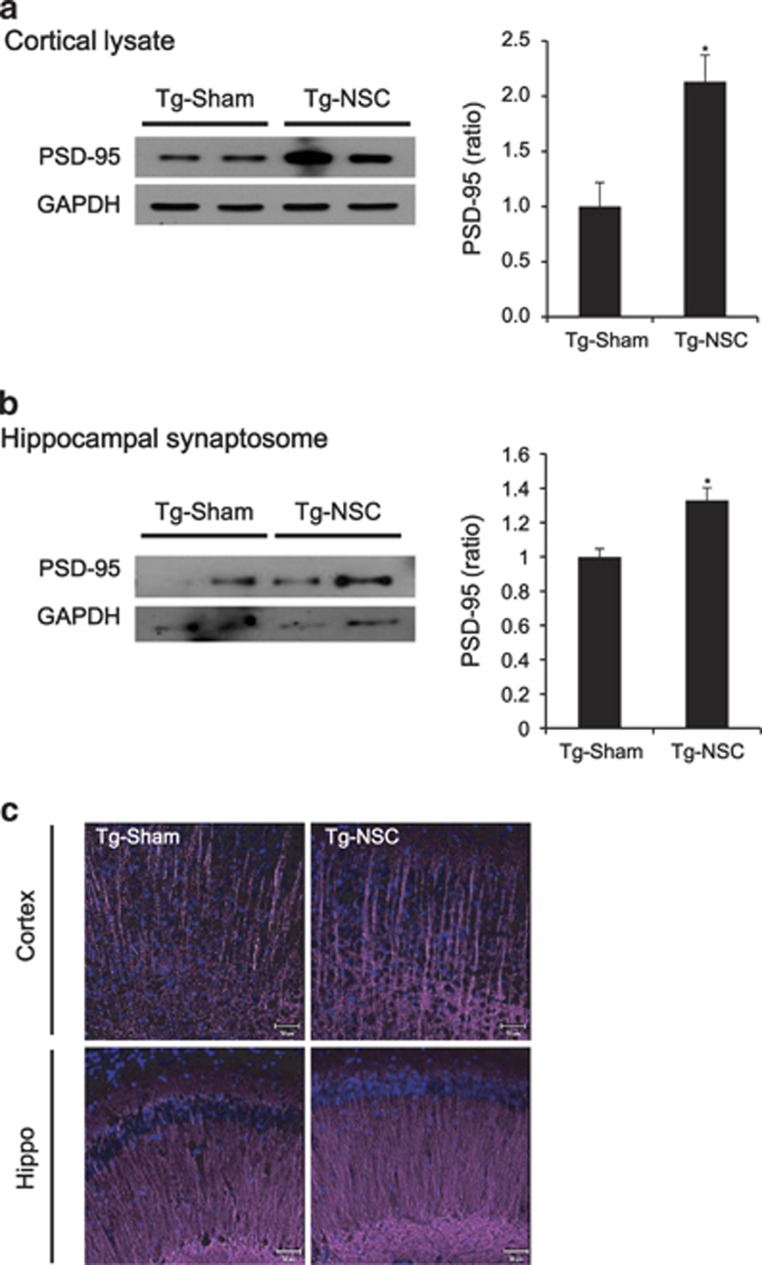
Synaptic density was increased in the NSC-transplanted Tg2576 mice. (**a** and **b**) PSD-95 expression level was analyzed by western blot. The level of PSD-95 was increased in the cerebral cortical area of Tg-NSC mice brains at 2.5 months after the transplantation compared with the Tg-sham group (**a**). In hippocampal synaptosomal fractions, PSD-95 level of Tg-NSC mice brains was increased compared with the Tg-sham mice brains (**b**). Quantitative data for PSD-95 are expressed as mean ±S.E.M. *P<0.05, by one-way ANOVA. (**c**) At 2.5 months after NSC transplantation, progressive reduction in MAP2 immunoreactivity was observed in the cortex and CA1 area of Tg-sham group mice. However, dendritic spine morphology was repaired and the density was increased by NSC treatment

We next performed immunolabeling studies to explain the functional recovery in the Tg-NSC group and focused on the possible links between neurogenesis and synapse formation. Postsynaptic density protein 95 (PSD-95) is an important factor that contributes to synaptic formation, and has been proposed to be a molecular scaffold for receptors and the cytoskeleton in synapses.^33^ Initially, we checked the PSD-95 levels in the cortex and hippocampus of the brains. In the brains of NSC-transplanted Tg2576 mice, PSD-95 was increased 2.1-fold (*P*<0.05) in cortical brain lysates (Figure 6a) and increased 1.3-fold (*P*<0.05) in hippocampal synaptosome fractions (Figure 6b) compared with the Tg-sham group. Immunostained images showed the lower intensity of PSD-95 in the hippocampus, especially DG, of Tg-sham mice brains compared with other group mice brains (Supplementary Figure 5).

In addition, we compared the morphology of neuronal dendrites by immunohistochemistry using the MAP2 antibody. MAP2-positive dendritic shafts were sparsely distributed and often fragmented in the brains of Tg2576 mice (Figure 6c). Brain sections were also triple-labeled using anti-Iba-1 antibody for microglia, thioflavin S staining for A*β* deposition and MAP2 for neuronal dendrites. As shown in Supplementary Figure 3, A*β* deposition surrounded with microglia seems to interfere with the processing of neuronal dendrites and induce the fragmentation of neuronal dendrites in the brains of Tg2576 mice. The number and size of A*β* deposition and microglial activation may affect the dendrite stability. However, more straight and prominent dendrites were densely distributed in the Tg-NSC group (Figure 6c), possibly indicating dendritic hypertrophy induced by NSC transplantation.

Together, these data suggest that NSC transplantation at the early stage of AD may have potential benefits of restoring both structural and functional deficits though enhanced endogenous neurogenesis and improved synapto-dendritic architecture (Supplementary Figure 6).

## Discussion

AD is characterized by massive neuronal death and synaptic disruption because of amyloid plaques and fibrillary tangles in several brain regions. Therefore, extensive recovery is necessary for AD therapy. Recently, many groups have suggested that stem cell transplantation could have therapeutic potential for AD. In contrast to early studies that mainly focused on the replacement of dead cells, more recent studies focus on understanding the diverse effects of stem cells. Some studies have shown that BMSCs trigger immune/inflammation responses through the activation of microglia in APP/presenilin 1 (PS1) double-transgenic (Tg) mice.^17, 19^ Other studies suggest that stem cells can induce neurotrophic factors.^10, 14, 15^ Indeed, BDNF was essential for the behavioral effects of NSC in triple Tg mice (3xTg-AD).^34^

In this study, we found that the NSC transplantation at an early stage of the disease reduced memory deficits, amyloid plaque load and tau pathology in AD model mouse, Tg2576. For these positive effects of stem cell transplantation, we provided two possible mechanisms: modulation in the processing of APP and the clearance of A*β* peptides, and the promotion of neurogenesis and synapse formation.

Owing to the progressive and broadly affected characteristics of AD, we examined the age of animals receiving transplants. In Tg2576 mice, a rapid increase of A*β* started at 6 months, amyloid plaques were formed at 9–12 months and memory deficits began from 12 months. In this study, we bilaterally transplanted NSCs into the DG of the hippocampus and the third ventricle of 12-month-old (early stage of AD) or 15-month-old (advanced stage of AD) Tg2576 mice, and age-matched WT mice. In the 15-month-old Tg2576 mice, the transplantation of NSCs did not exert a beneficial pathological or behavioral effect. When we transplanted NSCs into 12-month-old Tg2576 and WT mice, learning and memory were significantly improved. The number of amyloid plaques and phosphorylated tau were decreased in the brains of the Tg-NSC mice. These results indicated that the prevention of the disease progression at the early stages is important in AD therapy. Therefore, we suggest that it is essential to find a suitable period for the application of stem cells to obtain the best therapeutic effects of stem cell transplantation and some mechanisms of stem cell effects.

Our data indicated that the transplantation of NSCs at an early stage of the disease decreased the level of A*β* peptides and the amyloid plaque density induced in Tg2576 mice. In addition, we found an increase of the neprilysin levels and a reduction of BACE activity. Some studies have shown that the microglia secrete proteolytic enzymes, such as neprilysin, insulin-degrading enzyme (IDE), matrix metalloproteinase 9 (MMP-9) and plasminogen.^24, 35, 36^ This microglial behavior promotes A*β* clearance and may be helpful for inhibiting neurodegeneration in an AD brain. In other studies, glial activation induced by proinflammatory cytokines subsequently upregulated the expression level and enzyme activity of *β*-secretase, which can be inhibited by anti-inflammatory drugs.^37, 38^ A recent report has shown that the microglia from an aging AD model mouse reduced the expression of A*β*-degrading enzymes; thus, these microglia become defective in their ability to degrade A*β*.^39^ However, our immunohistochemical data showed the presence of many microglias in the brains of the Tg-sham or Tg-NSC groups, but A*β* plaques were still present in the brains of the Tg-sham group, which was in contrast to the in Tg-NSC group. This could be explained with the changed characteristics of microglia in aging AD model mice. In addition, secreted cytokine profiles were changed after NSC transplantation; the proinflammatory cytokine, IL-1*β*, was decreased and the anti-inflammatory cytokine, IL-10, was increased. Therefore, it would be plausible that the reduction of A*β* was because of the phagocytic capacity of microglia; changes in the production of cytokines affect the expression of neprilysin and *β*-secretase activity. Therefore, we suggest that NSC transplantation altered the microglial characteristics, leading to a change in the cytokine production profiles in the brains, and microglia may modulate the APP processing and A*β* clearance capabilities through the induction of neprilysin and the decrease of BACE activity. In the A*β* clearance by microglia, an important factor may be the characteristics of the microglia rather than their number.

Our previous study showed that intravenous or intracerebral transplantation of ASCs significantly improved learning and memory and restored neuropathology including A*β* deposition in Tg2576 mice in the same manner with NSC.^18^ In ASC-transplanted Tg mice brains, IL-10 and VEGF were upregulated.^18^

Although we showed the decline of amyloid plaques, additional lines of evidence are required to determine the stem cell transplantation-mediated cognitive improvements in AD models. As age increases, the production of new neurons was decreased, which is associated with memory decline,^40, 41, 42^ suggesting a connection between the failure of adult hippocampal neurogenesis and memory loss in AD. In our study, we examined hippocampal neurogenesis using immunostaining with anti-DCX antibody, and these DCX-positive cells did not colocalize with the transplanted NSC detected by anti-BrdU antibody or GFP-lentivirus; thus, DCX-positive cells represent endogenous neurogenesis. Interestingly, we also found an increased level of PSA-NCAM, which provides additional molecular evidence for increasing the endogenous neurogenesis.^43^

Some reports have shown that active microglia can be beneficial by producing anti-inflammatory cytokines and enhancing neurogenesis.^44^ In addition to the effect of microglia, VEGF has also been reported to exert neuroprotective and neurogenic effects,^45^ which was similar to other growth and neurotrophic factors. In this study, the levels of anti-inflammatory cytokines and VEGF were increased in NSC-engrafted mouse brains. Therefore, the increased endogenous neurogenesis could be explained by the neuroprotective microglia and VEGF.

In conjunction with the cognitive impairment, the significant loss of synapses and the cytoskeletal alteration occur at the early clinical stages of AD. Finally, we confirmed the recovery of brain function by assessing synaptic proteins and dendritic morphology. Experience-dependent synapse remodeling underlies learning and memory. Therefore, the impairment of synaptic plasticity is considered to cause cognitive deficits in an AD model mice.^21^ Indeed, PSD-95 deficiency was reported in Tg2576 mouse brains.^46, 47^ Along with PSD-95, MAP2 levels were enhanced by NSC transplantation, suggesting an increase of dendritic complexity. Tau is a microtubule-associated protein (MAP) that functions to stabilize microtubules. However, tau is highly phosphorylated in AD, leading to the formation of neurofibrillary tangles. The phosphorylation of tau tends to weaken the tau–microtubule interaction and disrupt the neuronal networks. For this reason, short dendrites more frequently may be observed in the brains of Tg2576 mice. However, after NSC transplantation, MAP2-stained dendrites were much longer and densely located in the cortex and hippocampal CA1 regions of Tg2576 mice. Together with the reduction of phospho tau protein, microtubule stabilization and synapse formation were promoted by NSC transplantation.

Although transplanted NSCs were rarely observed at 3 months after transplantation, the effects of transplantation were extensive. Following the alterations in cytokine production, first, the A*β* burden was diminished by changing the APP processing and inducing the degrading enzyme. Second, endogenous neurogenesis and neurogenesis-related proteins were increased. Third, the restorations in synapto-dendritic architecture influenced the improvement of learning and memory in Tg2576 mice.

These results clearly show that transplantation of NSCs at the early stages of the disease in Tg2576 mice could block the progress of AD pathology and memory loss in Tg2576 mice, but transplantation at an advanced stage could not.

## Materials and Methods

### NSC culture

NSCs are derived from the fetal (13 days) cerebral cortex of 6- to 7-week-old pregnant C57BL/6 mice. NSCs were cultured in Dulbecco's modified Eagle's medium/F-12 (1 : 1) (Gibco, Grand Island, NY, USA) medium supplemented with 2 mmol/l L-glutamine (Gibco), 0.6% glucose, 5 *μ*mol/l HEPES, 25 *μ*g/ml insulin, 100 *μ*g/ml apotransferrin, 30 nmol/l sodium selenite, 100 nmol/l putresine, and 20 nmol/l progesterone (all supplements purchased from Sigma, St. Louis, MO, USA) with growth factors of 10 ng/ml recombinant basic fibroblast growth factor (Roche, Mannheim, Germany) and 20 ng/ml epidermal growth factor (EGF; BD Sciences, San Jose, CA, USA).

### Transplantation

NSCs or media were transplanted to 12 or 15-month-old Tg2576 and age-matched WT mice. In all, 2 *μ*l of the cell suspension (within 1.5 × 10^5^ NSCs) were stereotaxically injected into DG of bilateral hippocampus (AP, −0.14 mm; ML, ±0.13 mm; DV, −0.19 mm) and the third ventricle (AP, −0.02 mm; DV, −0.35 mm) using a Kopf stereotaxic frame (Kopf Instruments, Tujunga, CA, USA) (Supplementary Figure 1). The injection rate was 0.4 *μ*l/min for 5 min and retracted after 5 min. Implantation surgery was performed under anesthesia with Rompun and Zoletil (1 *μ*g/g, i.p.). After the surgery, each mouse was kept in an individual cage.

### Behavioral test

We performed the Morris water maze at 2 months after the transplantation to measure spatial reference learning and memory based on the previously described method.^48^ A training session consisted of a series of three trials per day for 6 consecutive days and a single probe trial was conducted 48 h after the final training session,

### Histological examination

#### Immunohistochemistry

The mice were anesthetized with Rompun and Zoletil (1 *μ*g/g, i.p.) and transcardially perfused by cold saline with heparin after the conduction of behavioral tests. All the hemispheres were fixed overnight in 4% paraformaldehyde at 4 °C, antigen retrieved overnight in 0.01 M citric acid at 4 °C and boiled within citric acid then dehydrated in 30% sucrose and stored at 4 °C. In all, 25 *μ*m coronal sections of fixed hemispheres were cut through the entire hippocampus using a cryotome (Thermo Electron Corporation, Waltham, MA, USA) and stored in cryopreserve solution (30% glycerin, 30% ethylene glycol in 0.2 M PBS, pH7.4). Sections were stained by free-floating staining method. Sections were first retrieved by 0.01 M citric acid (pH 6.0) and blocked with 0.5% triton X 100 and 2% normal serum in TBS, then incubated with primary antibody in blocking solution overnight at 4 °C. For visualization under confocal microscopy, fluorescence-conjugated secondary antibodies were incubated for 1 h at RT. After immunostaining, specimens were examined on Zeiss LSM 510 confocal imaging system (Zeiss, Heidelberg, Germany).

#### Congo red and hematoxylin staining

Hydrated sections were incubated in hematoxylin (DakoCytomation, Glostrap, Denmark) for 10 min and destained by dipping five times in 1% HCl in 80% ethyl alcohol. After washing with running tap water, sections were stained with Congo red for 10 min at room temperature. And then sections were rinsed through ascending grades of ethanol with a final three changes of 100% reagent grade ethanol, cleared in xylene and cover slipped Canadian balsam solution.

### Synaptosome preparation

Synaptosomes were prepared from hippocampi using a volume-adjusted protocol established for whole brains. Briefly, the tissue was homogenized in 1 ml sucrose buffer (SB; 0.32 M sucrose, 1 mM NaHCO_3_, 1 mM MgCl2, 0.5 mM CaCl_2_) with homogenizer by 12 strokes at 700 r.p.m. on ice. Cell debris and nuclei were pelleted twice by centrifugation at 1400 × *g* for 10 min at 4 °C. The supernatants were cleared by centrifugation at 720 × *g* for 10 min at 4 °C, and then centrifuged at 13 800 × *g* for 10 min at 4 °C. The pellet was resuspended in 300 ml SB, layered over 1 ml pre-cooled 5% Ficoll and centrifuged at 45 000 × *g* for 45 min at 4 °C. The pellet was resuspended in 100 ml pre-cooled 5% Ficoll, which was then layered over 1 ml pre-cooled 13% Ficoll and centrifuged at 45 000 × *g* for 45 min at 4 °C. An interface containing synaptosomes was diluted with SB and centrifuged at 45 000 × *g* for 45 min at 4 °C to pellet the synaptosomes, followed by resuspension in 40 mM Tris-HCl (pH 6), 2% TritonX-100, 0.5 Mm CaCl_2_ containing protease inhibitors, incubated for 15 min at 4 °C and then centrifuged at 40 000 × *g* for 30 min at 4 °C. Pellet was washed with same buffer and then was resuspended in 20 mM Tris-HCl (pH8), 100 mM NaCl, 1 mM EGTA, 1 mM EDTA, 0.5% sodiumdeoxycholate, 0.1% SDS, 1% TritonX-100, containing protease inhibitors. After incubation for 15 min at 4 °C, samples were centrifuged at 40 000 × *g* for 30 min at 4 °C. Pellet was resuspended in 5% SDS and cleared it by centrifugation at 20 000 × *g* for 10 min at 4 °C.

### Western blot

Total proteins were extracted from the half of brain by homogenization in RIPA buffer with cocktail of protease inhibitors (Roche). Proteins were separated by SDS-PAGE or Tris/Tricine gel and transferred to a PVDF membrane. The PVDF membrane was blocked with 5% nonfat dry milk in Tris-buffered saline. After 1-h blocking, the protein blot was confirmed with appropriate antibodies and detected using horseradish peroxidase-conjugated secondary antibody (Amersham Pharmacia, Piscataway, NJ, USA).

### ELISA

ELISAs were performed using colorimetric sandwich ELISAs kits (A*β*42 ELISA: Invitrogen (Carlsbad, CA, USA), IL-1*β*: Biosource International (Camarilo, CA, USA), IL-10: Invitrogen) for the quantitative determination of A*β*42, IL-1*β* and IL-10 in brains. All assays were performed according to manufacturer's specific instructions. Levels of these proteins were calculated from a standard curve developed with specific OD *versus* serial dilutions of known concentration. Each standard and experimental sample was run in duplicate and the results were averaged.

### Secretase activity test

The fluorometric assay of secretase was conducted using *β*- and *γ*-secretase activity kits (R&D Systems, Inc., Minneapolis, MN, USA) in accordance with the protocol supplied by the manufacturer. As an enzyme source, total cortical protein lysates were tested. Quantification of substrate cleavage was assessed using a fluorometric reader (355 nm excitation, 510 nm emission).

### Antibodies

Primary antibodies were used as follows: goat anti-DCX (1 : 100; Santa Cruz, Dallas, TX, USA), mouse anti-MAP2 (1 : 100; Millipore, Billerica, MA, USA), rabbit anti-Iba1 (1 : 2000; Wako, Richmond, VA, USA), mouse anti-6E10 (1 : 1000, Covance, San Diego, CA, USA), mouse anti-phospho tau (1 : 1000, Pierce, Rockford, IL, USA), goat anti-tau (C17) (1 : 1000, Santa Cruz), rat anti-neprilysin (1 : 500, R&D Systems, Inc.), mouse anti PSA-NCAM (1 : 2000, Millipore), rabbit anti-VEGF (1 : 1000, Santa Cruz), mouse anti-PSD-95 (1 : 2000, Thermo Scientific, Waltham, MA, USA), rabbit anti-glyceraldehyde 3-phosphate dehydrogenase (GAPDH; 1 : 10000, Ab Frontier, Seoul, South Korea).

### Statistical analysis

Data were expressed as mean ±S.E.M. value or as ration of control value±S.E.M. Statistical analysis was performed by the one-way ANOVA: Tukey's HSD *Post Hoc* test using PASW statistics (SPSS version 18, IBM, Armonk, NY, USA). The difference was considered statistically significant for *P≤0.05, **P≤0.01 and ***P≤0.001.

## Abbreviations

A*β*: amyloid beta
AD: Alzheimer's disease
APP: amyloid precursor protein
ASCs: adipose-derived stem cells
BDNF: brain-derived neurotrophic factor
BMSCs: bone marrow stem cells
BrdU: bromodeoxyuridine
CTFs: carboxy-terminal fragments of APP
DCX: doublecortin
DG: dentate gyrus
EGF: epidermal growth factor
GDNF: glial-derived neurotrophic factor
GAPDH: glyceraldehyde 3-phosphate dehydrogenase
IDE: insulin-degrading enzyme
IGF: Insulin-like growth factor
IL-1*β*: interleukin-1*β*
IL-10: interleukin-10
MAP2: microtubule-associated protein-2
MMP-9: matrix metalloproteinase
NGF: nerve growth factor
NSCs: neural stem cells
PS1: presenilin 1
PSA-NCAM: polysialylated-neural cell adhesion molecule
PSD-95: postsynaptic density protein 95
Tg: transgenic
Tg-sham: saline injected transgenic mice
Tg-NSC: NSCs transplanted transgenic mice
VEGF: vascular endothelial growth factor
WT: wild type
WT-sham: saline injected WT mice
WT-NSC: NSCs transplanted WT mice
