# Supplementary material for: Neural stem cell transplantation at critical period improves learning and memory through restoring synaptic impairment in Alzheimer's disease mouse model
Source: Cell Death Dis. 2015 Jun 18;6(6):e1789–. doi: 10.1038/cddis.2015.138 (PMC4669825; doi:10.1038/cddis.2015.138)
Supplement: Supplementary Figure Legends [file cddis2015138x1.doc]

**Supplementary Figure Legends**

**Supplementary Fig 1. Experimental scheme of neural stem cell transplantation**

(A) The injection sites of NSC transplantation. The NSCs were stereotaxically transplanted into the dentate gurus of bilateral hippocampus (AP, -0.14mm; ML, ±0.13mm; DV, -0.19mm) and the third ventricle (AP, -0.02mm; DV, -0.35mm). (B) NSCs or culture media were injected to 13- or 15-months-old Tg2576 or age matched wild type mice. 2 months after the surgery (15- or 17-months old), behavioral tests were conducted for 2 weeks (0.5 months), and then the mice were sacrificed. To examine the early stage of transplantation, mice were sacrificed 3 weeks after the surgery.

**Supplementary Fig 2. NSC transplantation decreased the number of Congo red stained amyloid plaques and γ –secretase enzymatic activity in Tg2576 mice.**

(A) 2.5 months after the transplantation, the number of amyloid plaques stained by Congo red was dramatically reduced, especially in frontal, entorhinal and piriform cortex in the cortex of Tg-NSC mice brains. (B) γ–secretase enzymatic activity was assessed in cortical lysates by fluorometric reaction. γ–secretase activity was increased in the cortex of Tg-sham mice brains; however, it was reduced by NSC transplantation, but it was not statistically significant. *p<0.05, **p<0.01, ***p<0.001 by one-way ANOVA.

**Supplementary Fig 3. Amyloid plaques and microglial cells in cortex and hippocampal regions of Tg2576 mice brains**

In triple staining of thioflavin S, IbaI, and MAP2, amyloid plaques and microglial cells in cortex and hippocampal regions were detected and microglial cells were gathered around the plaques. Dendrites stained by MAP2 were increased even in the area within the amyloid plaques in Tg-NSC group brains.

**Supplementary Fig 4.** **Endogenous neurogenesis was increased in the NSC transplanted Tg2576 mice.**

Endogenous neurogenesis was observed in the dentate gyrus of hippocampus by immunohistochemisty using anti-BrdU antibody. The number of BrdU-positive cells was calculated in dentate gyrus. Graph represents that endogenous neurogenesis was significantly enhanced at 2.5 months after NSC transplantation. *p<0.05, by T-test.

**Supplementary Fig 5.** **Endogenous neurogenesis was increased in the NSC transplanted Tg2576 mice.**

Immunohistochemistry of PSD-95 in layer 2/3 VC from Tg-sham show reductions in endogenous and immunostained PSD-95 puncta, but enhanced staining in Tg-NSC. Total pixel intensity of PSD-95-positive immunolabels was quantified in DG and CA3 area. Scale bar, 50μm & 100μm.

**Supplementary Fig 6.** **Putative mechanisms for the multiple actions of NSCs in Tg2576 mice brains.**
