# Supplementary figures and images for: Neural stem cell transplantation at critical period improves learning and memory through restoring synaptic impairment in Alzheimer's disease mouse model
Source: Cell Death Dis. 2015 Jun 18;6(6):e1789–. doi: 10.1038/cddis.2015.138 (PMC4669825; doi:10.1038/cddis.2015.138)

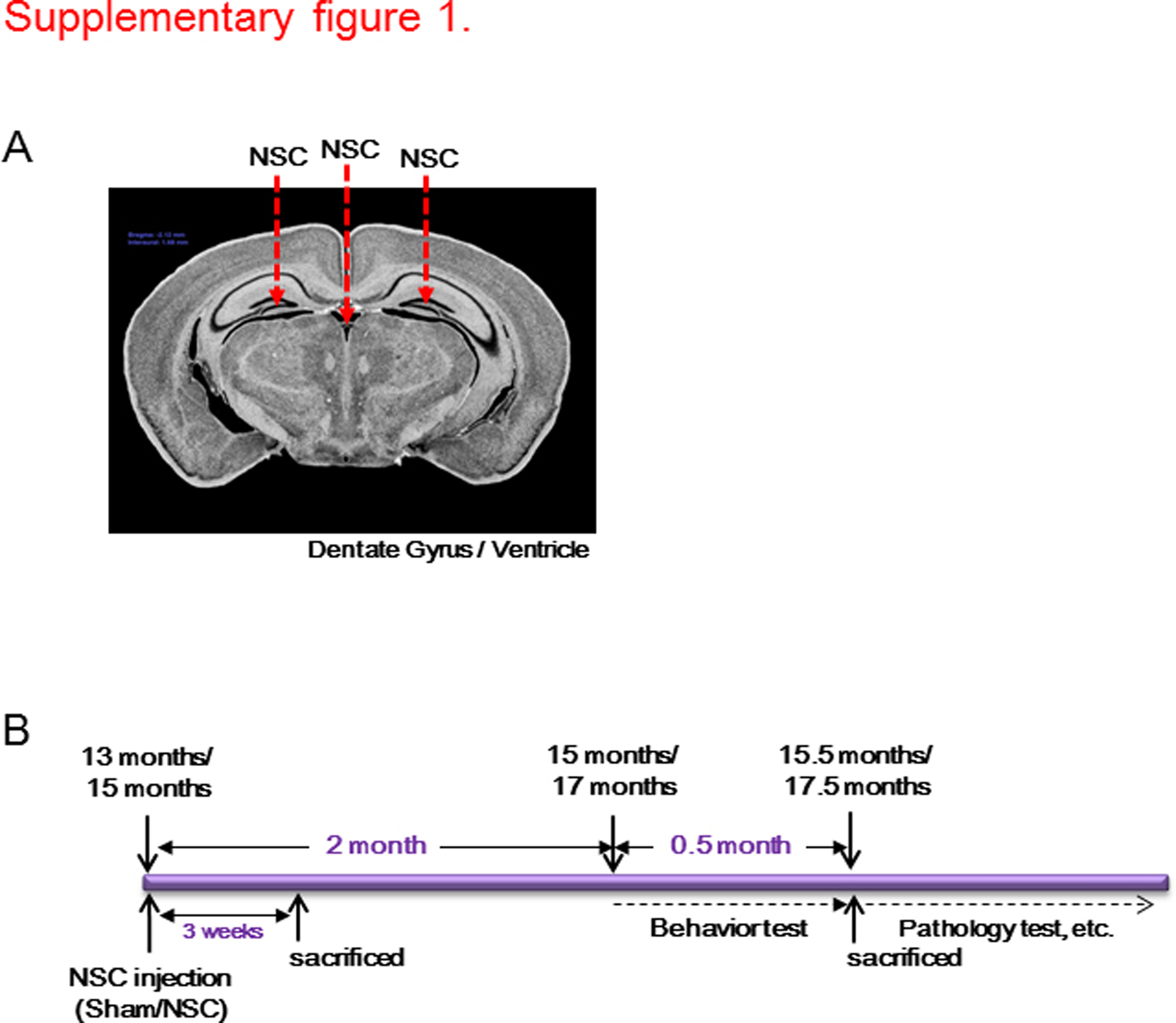

Supplement: Supplementary Figure 1 [file cddis2015138x2.tif]

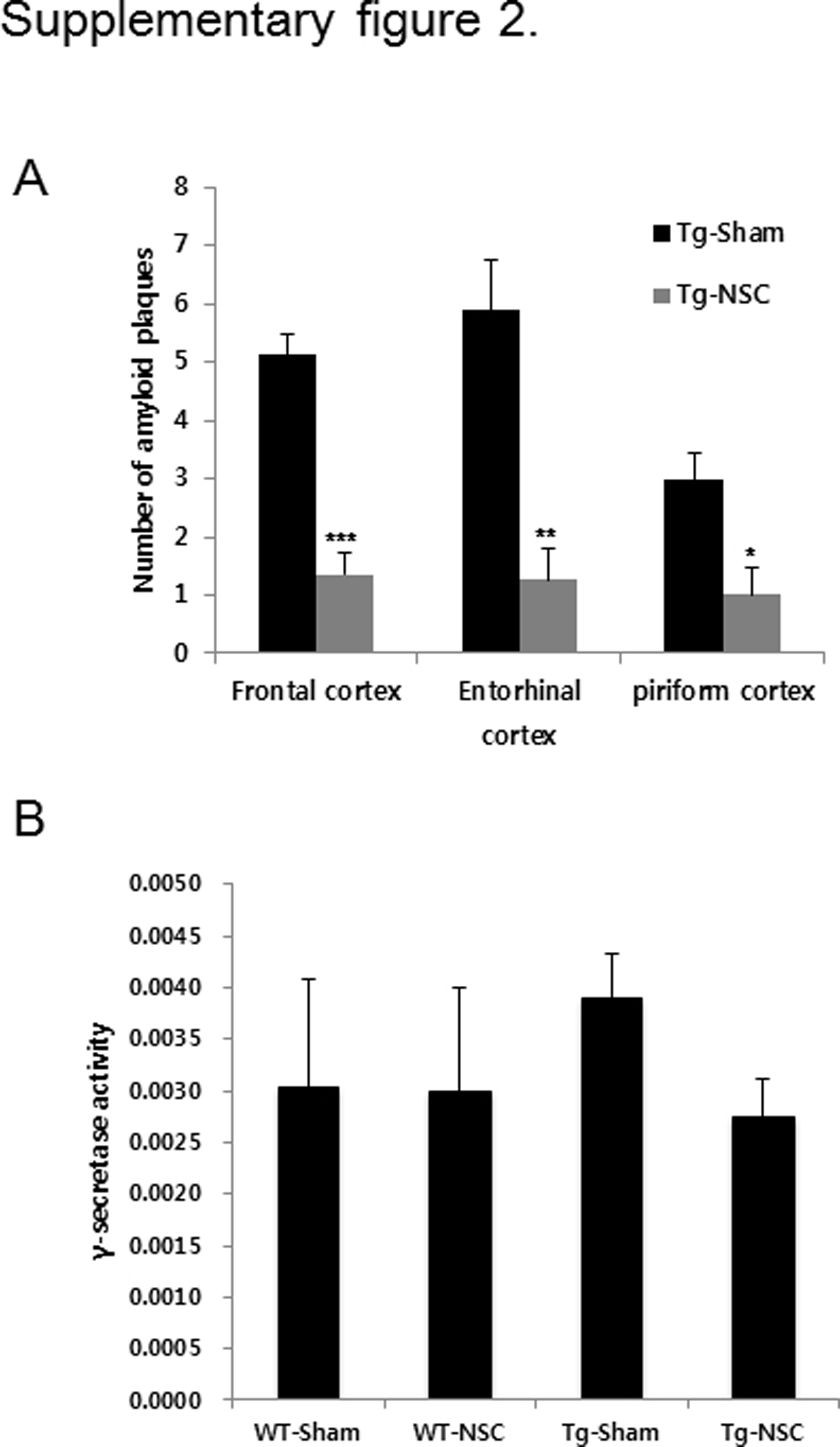

Supplement: Supplementary Figure 2 [file cddis2015138x3.tif]

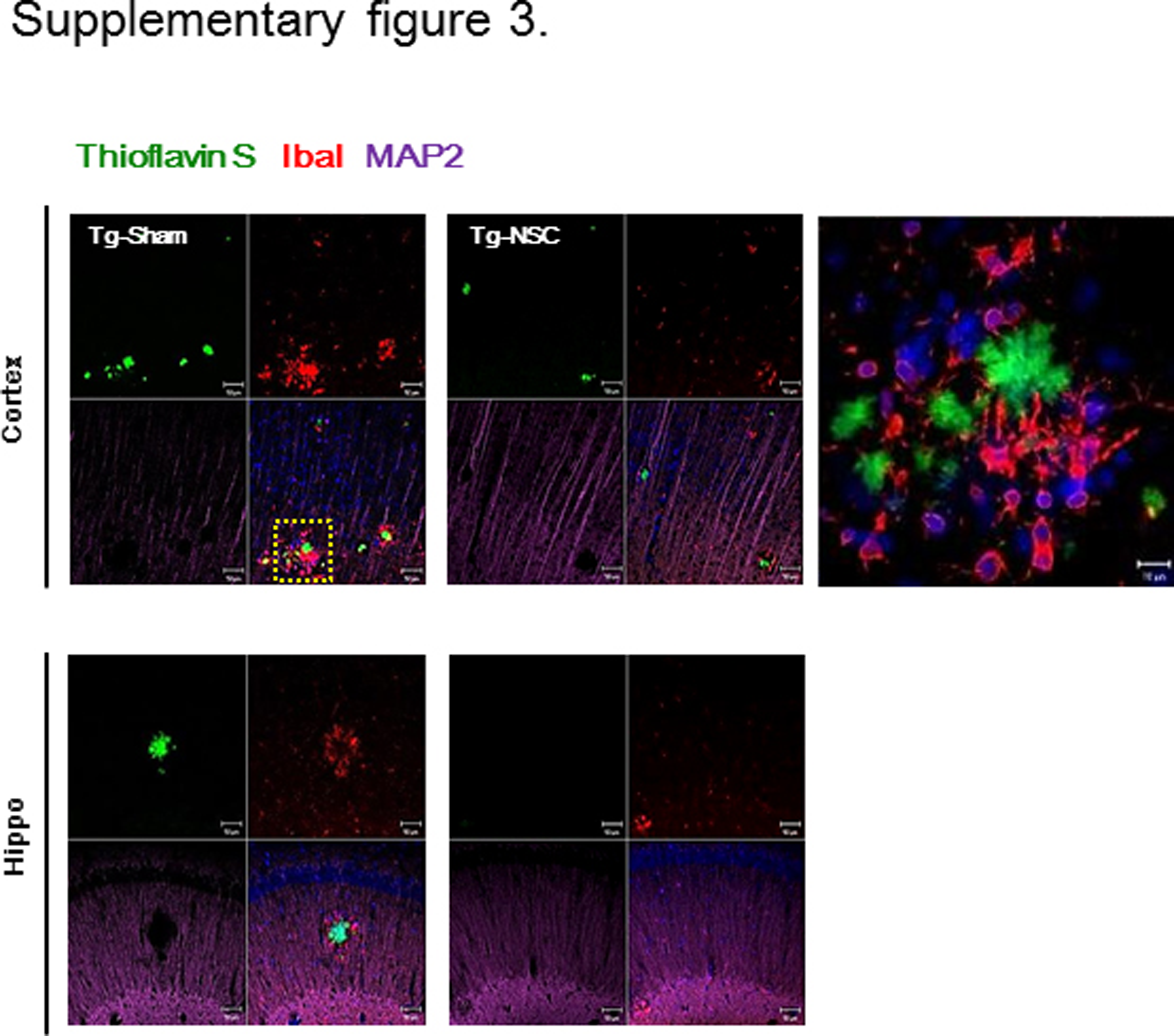

Supplement: Supplementary Figure 3 [file cddis2015138x4.tif]

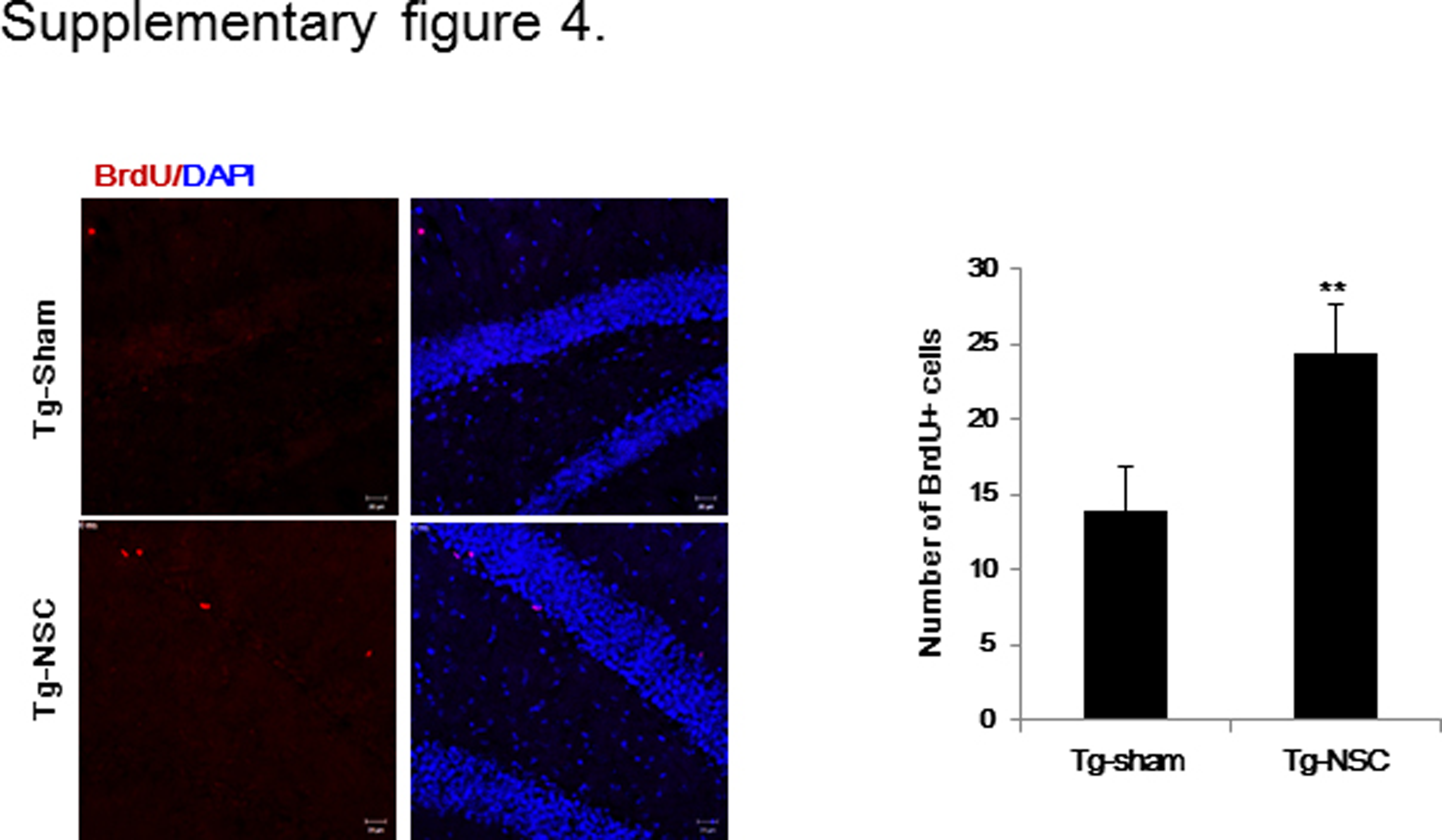

Supplement: Supplementary Figure 4 [file cddis2015138x5.tif]

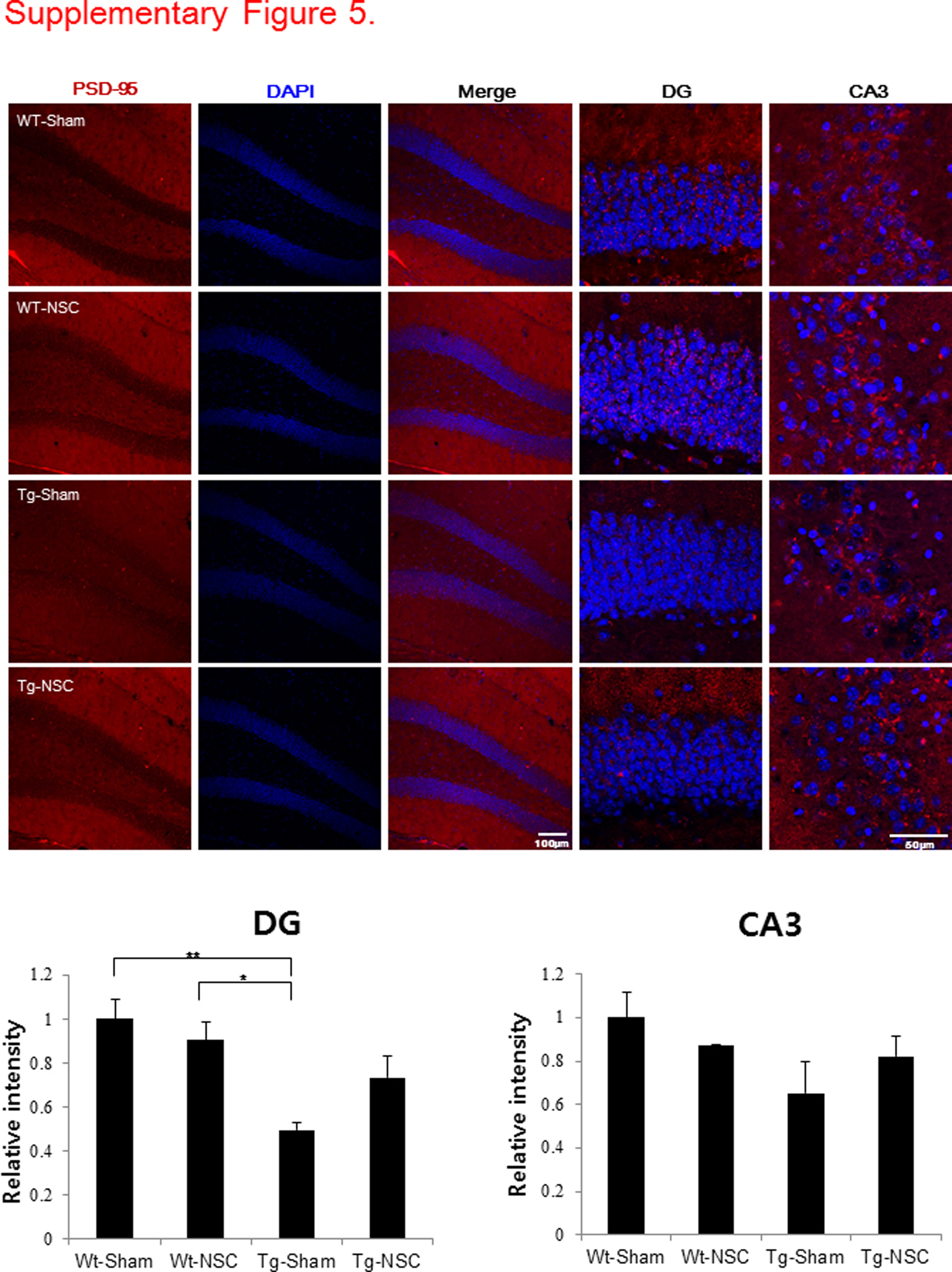

Supplement: Supplementary Figure 5 [file cddis2015138x6.tif]

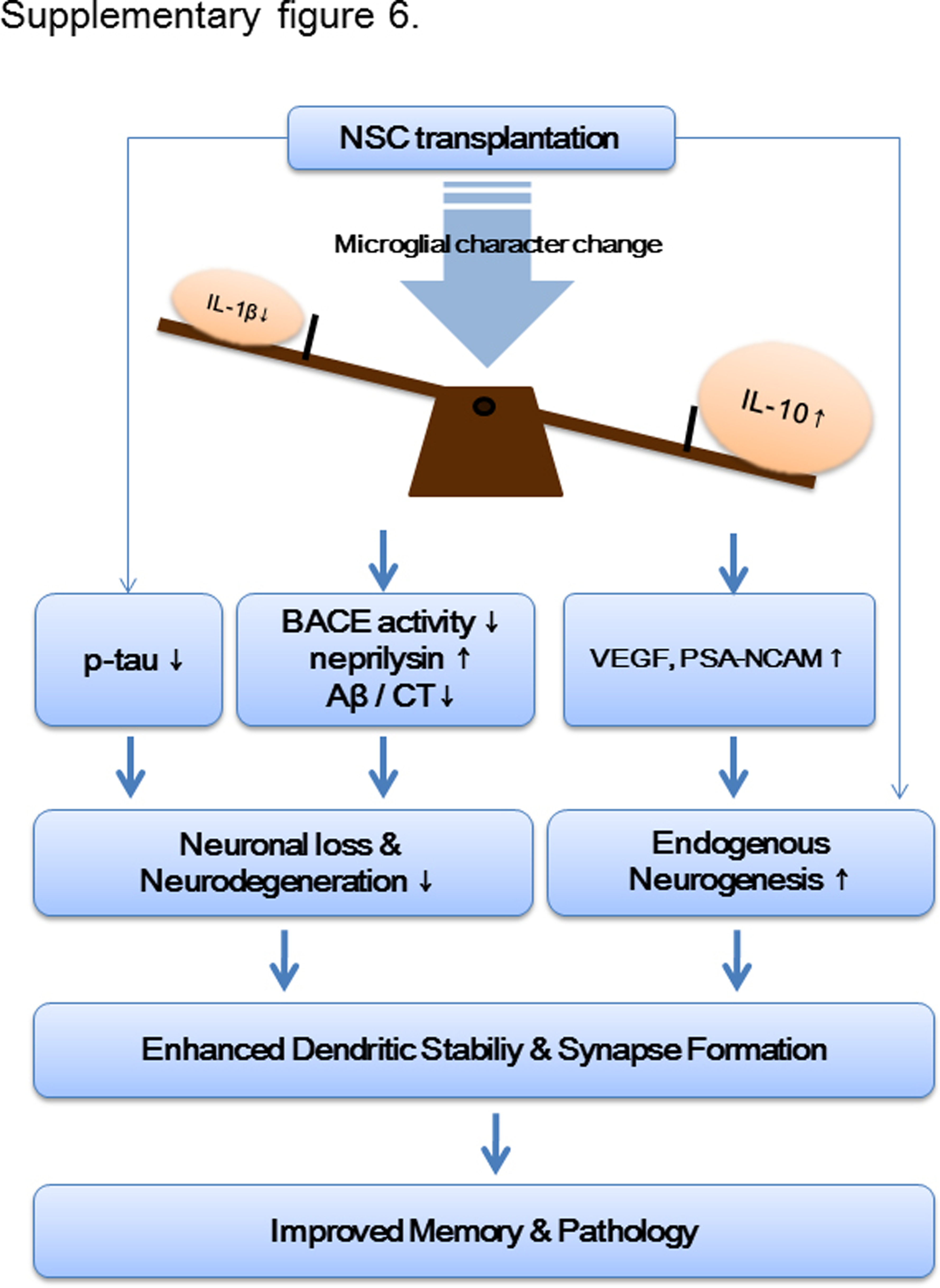

Supplement: Supplementary Figure 6 [file cddis2015138x7.tif]
